# Identifying the Current State and Improvement Opportunities in the Information Flows Necessary to Manage Professional Athletes: A Case Study in Rugby Union

**DOI:** 10.3389/fspor.2022.882516

**Published:** 2022-06-28

**Authors:** Jayamini Ranaweera, Dan Weaving, Marco Zanin, Gregory Roe

**Affiliations:** ^1^Carnegie Applied Rugby Research (CARR) Centre, Carnegie School of Sport, Leeds Beckett University, Leeds, United Kingdom; ^2^Bath Rugby Football Club, Bath, United Kingdom; ^3^Leeds Rhinos Rugby League Club, Leeds, United Kingdom

**Keywords:** player management, information modeling, sport informatics, Business Process Management, decision-making in sports

## Abstract

In sporting environments, the knowledge necessary to manage athletes is built on information flows associated with player management processes. In current literature, there are limited case studies available to illustrate how such information flows are optimized. Hence, as the first step of an optimization project, this study aimed to evaluate the current state and the improvement opportunities in the player management information flow executed within the High-Performance Unit (HPU) at a professional rugby union club in England. Guided by a Business Process Management framework, elicitation of the current process architecture illustrated the existence of 18 process units and two core process value chains relating to player management. From the identified processes, the HPU management team prioritized 7 processes for optimization. In-depth details on the current state (As-Is) of the selected processes were extracted from semi-structured, interview-based process discovery and were modeled using Business Process Model and Notation (BPMN) and Decision Model and Notation (DMN) standards. Results were presented for current issues in the information flow of the daily training load management process, identified through a thematic analysis conducted on the data obtained mainly from focus group discussions with the main stakeholders (physiotherapists, strength and conditioning coaches, and HPU management team) of the process. Specifically, the current state player management information flow in the HPU had issues relating to knowledge creation and process flexibility. Therefore, the results illustrate that requirements for information flow optimization within the considered environment exist in the transition from data to knowledge during the execution of player management decision-making processes.

## Introduction

In professional team sports environments, practitioners execute different operational processes to manage players. Such processes include managing an athlete's physiological, psychological, technical, and tactical preparation and performance (Jones et al., 2016; Sclafani and Davis, 2016; Tee et al., 2018). Due to modern technological enhancements (e.g., developments in computational power and miniaturization of electronics), staff executing such processes often rely heavily on the evidence generated from data and information to acquire the knowledge required to manage the players (Quarrie et al., 2016; West et al., 2019; Colomer et al., 2020). Originating initially from Ackoff's Data-Information-Knowledge-Wisdom (DIKW) hierarchy (Ackoff, 1989), Dammann has recently proposed a framework (Dammann, 2018) to provide a clear context to this transition among data, information, evidence, and knowledge from a health informatics perspective, and it appears to be directly relatable to sporting contexts. The latter framework suggests that data are raw symbols that become information when contextualized. Information compared to standards create evidence which can be used to test hypotheses that can transition to knowledge through success and consensus (Dammann, 2018). Wider information science research helps to further understand that this transformation from data to knowledge generally acts as a flow and can be referred to as an information flow (Scharmer, 1996).

From a practical viewpoint, previous rugby union research has presented the different information sources used by 12 Gallagher Premiership rugby union clubs in England to monitor and manage athletes (West et al., 2019). Such information sources act as the basis for generating evidence necessary for managing rugby union players in those professional environments. Athlete health management models proposed for professional sporting settings clearly illustrate that the decisions made relating to player management can involve multiple stakeholders (e.g., coaches and physicians) (Dijkstra et al., 2014). Hence, it may suggest that the transition from evidence to knowledge creation required for player management could primarily occur based on the consensus generated from collective decision-making processes. However, in applied sporting environments, there could be instances where the information flows may not be in an optimized state during player management. Such situations could be created due to the lack of data, information, and evidence quality, and from the sub-optimal transitioning between the different stages of the information flow. From an idealistic viewpoint, an optimized information flow for decision-making could possess high-quality data and information sources, have set standards for evidence generation, and be guided by a holistic knowledge management framework. Additionally, wider sports research illustrates the growing interest in utilizing available information streams to generate analytical models relating to athlete management (Beal et al., 2019; Claudino et al., 2019). However, while the development of such models may be beneficial and appealing, there are limited attempts to establish if the information flows feeding to those models and the wider player management processes are actually optimized prior to decision-making on a day-to-day basis. Formulating inferences from sub-optimal information flows could add additional noise to the decision processes and may result in incorrect judgments pertaining to player management. This could in turn risk the adequate preparation and performance of the athlete, highlighting the need to optimize the information flows associated with player management processes in sporting environments such as rugby union.

The first step to any optimisation task is to understand what specific aspects require improvement. Once the issues in the current state are identified, optimisation tasks can then be conducted to overcome those issues to derive a better future state. Therefore, at the macro-level, this article focuses on the first step to an information flow optimisation task by presenting a practical case study for determining the current state (illustrating the existing gaps and issues) of the player management information flow in the performance department of a professional rugby union environment. Specifically, the study aimed to identify and unravel solutions to the following set of questions: (1) in the performance department of a professional rugby union environment, what different processes are executed to manage the preparation and performance of the players? (2) in its current state, how does information flows within the identified processes? (3) are there any requirements to improve the current information flow of the identified processes?

## Methods

### Case Study

The present study evaluated the player management processes at a rugby union club competing in the Gallagher Premiership in England. During the 2019/2020 season, an organizational objective was set at the club to enhance the use of information for decision-making within the performance department or specifically referred to as the High-Performance Unit (HPU). Therefore, the current study is an outcome of specific management intent and presents the first part of the results obtained from a stepwise approach conducted to optimize the information requirements for player management processes in the HPU. As a business unit, the overall objective of the HPU was to maximize the availability of players in the squad to the rugby program at the club. At the time frame of the study, the HPU consisted of physiotherapists (5), strength and conditioning (S&C) coaches (3), sports scientists (1), doctors (1), medical administrators (1), and data scientists (1). Additionally, the head of medical, head of strength and conditioning, and head of applied sciences and research provided leadership to the HPU and will be referred to as the HPU management team throughout the article. Furthermore, the study was approved by the ethics committee of the affiliated university.

### Business Process Management Approach

**Figure 1:**
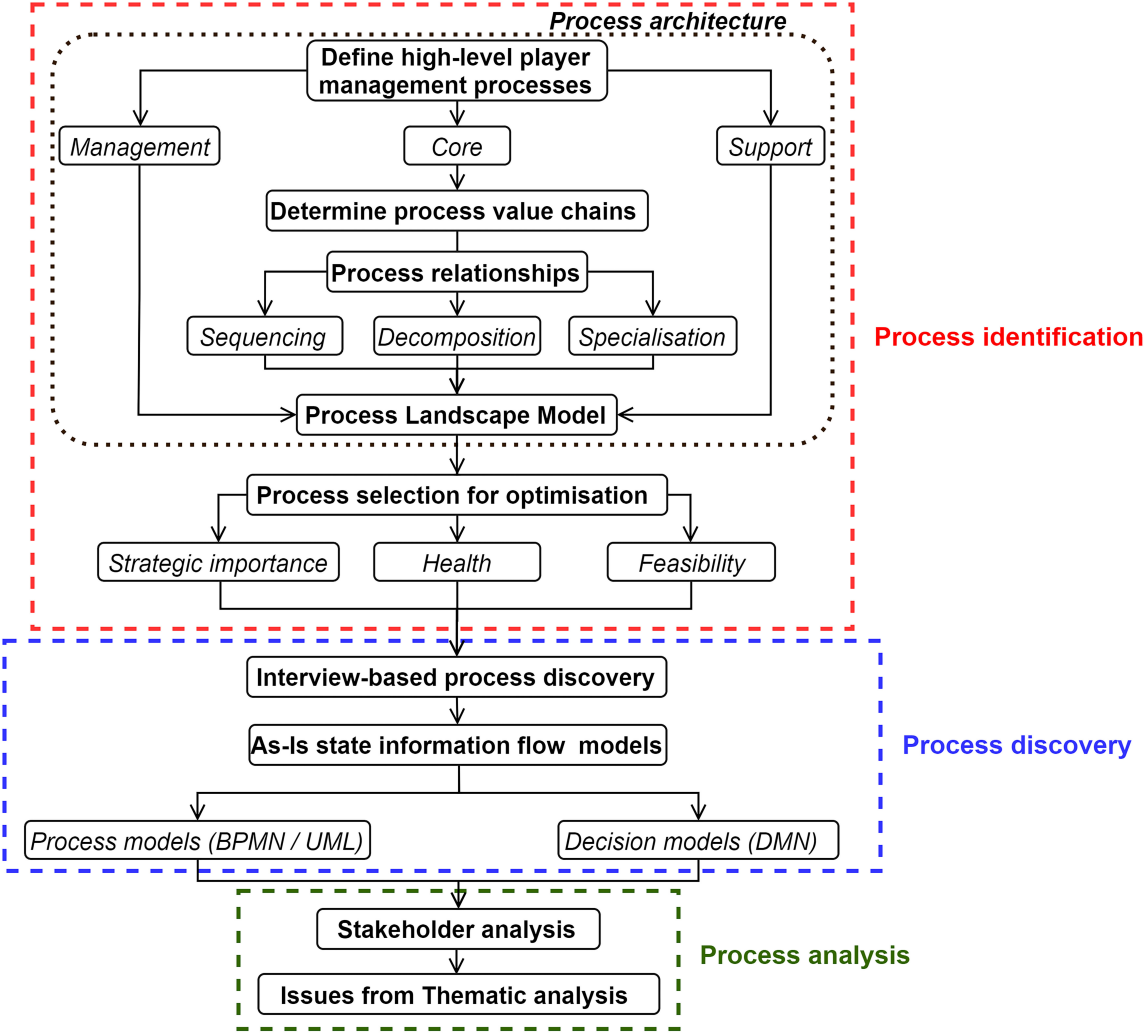
Steps to identify the current issues in the player management information flow.

In the current study, we adopted fundamentals from Business Process Management (BPM) (Dumas et al., 2018; Kirchmer et al., 2019) used within a wide array of service industries, such as finance, healthcare, banking, and information technology (Anand et al., 2013; Fernández et al., 2019), for determining the current state and improvement opportunities in the HPU information flow. As a change management tool, BPM has been successful in optimizing the information flows associated with service-oriented (intangible) processes, such as infection management (Cánovas-Segura et al., 2017). Since the nature of player management processes is service-oriented (e.g., physiotherapist providing soft tissue treatment, a coach providing technical knowledge to enhance player performance) and are supported by information pathways, BPM was considered as the appropriate framework to be utilized in the current study (Ranaweera et al., 2021). As introduced in Figure 1 and specified later, we used the first three phases (process identification, redesign, and analysis) of the BPM lifecycle presented by Dumas et al. (2018) as the framework to identify the issues existing in the current information flow within the High-Performance Unit.

- Process identification: Generates the organizational process architecture, performance measures, relationships and systematically identifies the processes that require a BPM intervention to assist in meeting organizational goals.
- Process discovery: For the identified processes, information on the current state (As-Is) is collected and documented through process modeling techniques.
- Process analysis: Issues within the documented As-Is state of the processes are identified for optimisation, potentially generating a list of prevailing issues.
- Process redesign: The process is redesigned (optimized) to overcome the issues identified in the process analysis stage to define the best future state (To-Be).
- Process implementation: Necessary changes to move the process from the As-Is to the To-Be state is performed by managing organizational changes.
- Process monitoring: The implemented process is monitored to determine the effectiveness of the changes. The cycle is repeated to the discovery stage if further issues are present or further continuous improvements are necessary.

### Process Identification

#### Process Architecture

The initial step to assessing the current state and identifying improvement opportunities within a player management information flow is to understand the different athlete management processes that occur in a specific environment. Technically, for the current study, this led to establishing the player management process architecture (Dumas et al., 2018) of the HPU. In a performance department, the process architecture corresponds to the group of inter-linked processes that cover most of the work executed by staff to manage the preparation and performance of the players. We have adopted a top-down approach (initiating from high-level processes and decomposing to small-scale executable processes) for deriving the process architecture of the HPU since it helped attain a broader view of the department than a bottom-up approach (Verner, 2004). Specifically, the high-level process architecture of the HPU was represented based on a process landscape model (Weske, 2007; Dumas et al., 2018), illustrating how the different player management processes were interconnected. The steps specified below were used to derive the process architecture of the HPU, and the role of every member was represented within a high-level process in the model.

- *Define high-level processes*: Author JR observed the execution of daily player management processes in the HPU for a period of 1 year (starting from December 2019) by taking a participant as an observer stance (Kawulich, 2005), where the author has been working as a member of the HPU. Observational data were initially recorded as instantaneously sampled field notes (Paolisso and Hames, 2010; White and Cicmil, 2016) and the higher-level individual player management processes were defined and segregated by the following three main process categories specified below (Dumas et al., 2018).
  ○ Core: Key player management execution processes in the HPU.
  ○ Management: Processes providing guidance for the execution of the core processes.
  ○ Support: Processes assisting for smooth execution of the core and management processes.
- *Identify the value chains:* As a definition, the value chains (Zamora, 2016; Dumas et al., 2018) in the HPU illustrated the set of core player management processes, which demonstrated a full distinct cycle of activities performed by staff to manage the players. In the given environment, the player management process execution sequence occurring within a typical week was used to define the links between the individual core player management processes and identify distinctive process value chains. Therefore, based on the observations, author JR identified the links (value chains) between the core processes which were defined previously and were temporarily recorded as process model sketches on paper (Trebble et al., 2010).
- *Process relationships:* The relationships between the individual processes in each core value chain were formulated based on decomposition, sequencing, and any specializations to derive the lower level sub-processes in the architecture (Dumas et al., 2018).
  ○ Decomposition: The process is described in more detail through one or more sub-processes.
  ○ Sequencing: There is a logical sequence between processes.
  ○ Specializations: There are several variants of a general process.
- *Process landscape model:* The defined core (including the value chains and excluding the relationships), management, and support processes were formulated into a process landscape diagram to demonstrate the high-level process architecture of the HPU.

The semantic quality (i.e., to understand how well the diagrams described the actual operation of the HPU) of the formulated process architecture was assessed by the HPU management team based on a 1-h meeting. The process units in the architecture comprised of processes generating an operational (i.e., providing a direct service to the player, team, or staff) or decision (i.e., generating a decision relating to the management of the player and/or team) outcome. From these two outcome types, the HPU management team decided to only concentrate on processes providing a decision outcome for identifying current issues in the information flow.

#### Process Selection

Since every organization has limited time and resources, it is not possible to evaluate the information flow of all the identified player management processes in a single attempt. Therefore, it was necessary to select the highest priority processes that required consideration. To achieve the latter objective, the HPU management team subjectively rated each of the identified process units using the below criteria (Dumas et al., 2018) during the former meeting.

- Strategic importance (high/low): To define the significance of each player management process to the current core strategy of the organization.
- Health (poor/good): Perspective on the current quality of the information flow associated with the considered process.
- Feasibility (low/medium/high): The feasibility of each process for change or optimisation. For example, if a process was associated with an information system that could not be altered (e.g., medical information system) or subjected to organizational politics, the feasibility was rated as low.

From the process ratings, processes having high strategic importance, with poor current information flow quality (health) and medium or high feasibility, were selected to be analyzed for current issues in the information flows associated with them.

#### Process Discovery

Once the set of player management processes in the HPU was identified and selected, it was possible to analyse in detail the current state (As-Is) of the information flow associated with those processes. The final goal of this stage was to derive the current process and decision-level models of the selected processes with clear indications of the information flow, specifically highlighting the points at which data, information, and knowledge were generated during process execution. The BPM literature suggests both automated and non-automated methods for process discovery (Jadhav, 2011). Automated methods include discovering the As-Is-state process models from information system event logs (Augusto et al., 2017), but this was not viable since player management processes in the HPU were more human-oriented rather than system-driven. Hence, we used interview-based process discovery (Verner, 2004) as a non-automated method to derive the in-detail process and decision models of the selected processes. For the latter purpose, the following three steps were adopted.

- *Data collection:* For data collection, practical guidelines available in the literature were utilized (Verner, 2004), and the following three key roles were defined prior to the interview sessions:
  ○ Sponsor: The individual chartering the overall optimisation project by defining the scope and goals. For the considered study, the head of sports science and research was defined as the sponsor.
  ○ Subject-Matter Expert (SME): The main individual(s) responsible for executing the identified player management process in the current context. Relevant SMEs for each process were defined by the sponsor with further feedback from other members of the HPU management team (specific details of the identified SMEs have been provided in the Results section).
  ○ Analyst: Responsible for collecting, organizing, analyzing, and presenting information regarding the process. Author JR acted as the analyst.

The analyst, with the presence of the sponsor, conducted semi-structured interviews (each <1 h) with the subject-matter experts (Table 4) to collect complete details necessary to model the current state (As-Is) information flow of the considered processes. The interview sessions were guided by the set of themes defined in Table 1, which were necessary to develop the detailed current state models of each process. Interview data were recorded sequentially for each theme during the interview.

- *Current state (As-Is) process models*: Information extracted from the process discovery interviews were translated into process models using collaboration and process diagrams in Business Process Model and Notation (BPMN), guided by the syntactic rules defined in the literature (Dumas et al., 2018) and international standards (ISO/IEC, 2013). Additionally, the decision points within the player management processes were modeled using decision requirement diagrams (Bazhenova et al., 2019) and decision tables (Calvanese et al., 2018) in Decision Modeling and Notation (DMN).
- *Process model quality assessment*: The semantic quality of the developed models was assessed by the SMEs and the sponsor. Prior to the quality assessment, author JR (analyst) explained the structural and behavioral rules used in the models to the relevant SMEs.

**Table 1:** Data collection themes for As-Is process discovery.

**Theme**	**Description**
Process name	Information on standard name of the process.
Process owner	Key individual assigned to a specific process and is responsible for developing, analyzing and continuously improving the process.
Process objective	What the process is intended to accomplish for the organization.
Trigger events	Events/tasks enabling execution of the analyzed process.
Actors	Main process participants and individuals affected due to the optimisation.
Information suppliers	Key individuals supplying information as inputs for process execution.
Information inputs	All relevant information inputs to the process.
Process steps	All steps involved in the process and their sequence of execution.
Information outputs	All relevant information outputs from the process.
Main customer	Key individuals receiving information as outputs from the process.
Process performance measure	Any measurements defined to evaluate the success of process execution.
Data management	How data are collected, analyzed and managed during process execution.
Technology	Any technology platforms used during process execution.

#### Process Analysis

Once the current state (As-Is) of the identified and prioritized processes in the HPU were modeled, it was important to identify the specific issues in the current information flow associated with those processes. Resolution of such issues will act as the basis for future optimisation tasks relating to the HPU information flow. The BPM literature provides different qualitative and quantitative approaches to process analysis (Dumas et al., 2018). Whilst qualitative methods like value-based analysis (i.e., to analyse which aspects of the information flow are value adding and non-value adding) have the potential to be beneficial in sporting contexts, the current sport literature does not provide enough evidence on how value is created within player management processes for sporting organizations. Therefore, stakeholder analysis (Burlton, 2010; Dumas et al., 2018) was deemed more suitable to analyse the current issues in the information flow of player management processes in the HPU. Specifically, stakeholder analysis focuses on identifying the different issues that exist in the current state of the process from the perceptions of the different stakeholders associated with it (e.g., customers, process owners, and process participants). The stakeholders of a process are generally aware of the process operating dynamics and mindful of the issues which exist in the information flow from their perspectives.

Hence, guided by the two question themes specified later, focus group discussions (each lasting 30–45 min) were conducted with the key stakeholder groups (e.g., physiotherapists, strength, and conditioning coaches) of the identified processes to elicit data on existing issues within the information flow of the discovered processes during player management. Participant details of those focus groups have been provided in the Results section (Table 6).

- Are there any issues with the data/information sources required for decision-making during the execution of the considered process?
- Are there any issues associated with transforming the available data to knowledge during the execution of the considered process (helped to determine noise in decision-making)?

Author JR acted as the moderator of these group discussions. Each session was conducted with the As-Is state model of the process as a reference material to guide the information elicitation process. Therefore, at the beginning of each session, author JR explained the BPMN diagrams (including the syntactic rules of BPMN) of the considered process to the participants.

Focus group data were collected as audio files for in-person sessions and video recordings for any virtual sessions. Thematic analysis was used to analyse the focus group data and was targeted at unraveling information on current issues within the information flow of the considered processes. This latter objective was achieved by using the six-phase thematic analysis method (Braun and Clarke, 2006) to surface themes of issues from stakeholder perspectives in the current information architecture. Guided by the five-step live coding method introduced for coding focus group data (Parameswaran et al., 2019), the coding structure and theme development focused on using semantic, latent, and inductive approaches (Virginia et al., 2016). Author JR initially conducted the thematic analysis process and validated the outcomes with authors GR and DW by reaching a consensus on the coding structure and the unraveled information flow issue themes. Next, the list of information flow issues in the current state was verified with the stakeholders of the considered processes. Finally, the results were shared with the management team at the club for further verification and feedback.

## Results

### Process Identification

#### Process Architecture

**Figure 2:**
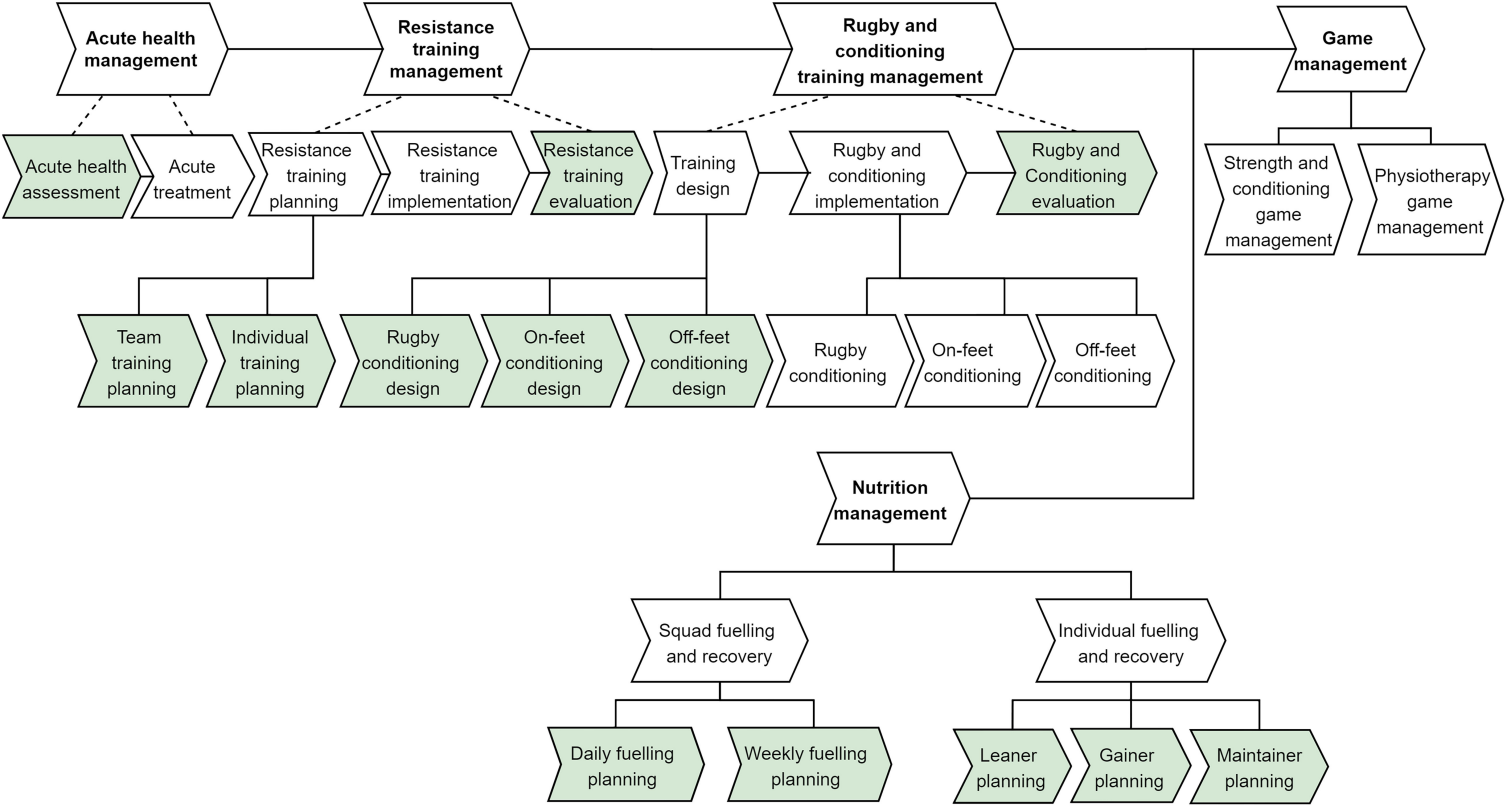
Process relationships in performance management core value chain.

**Figure 3:**
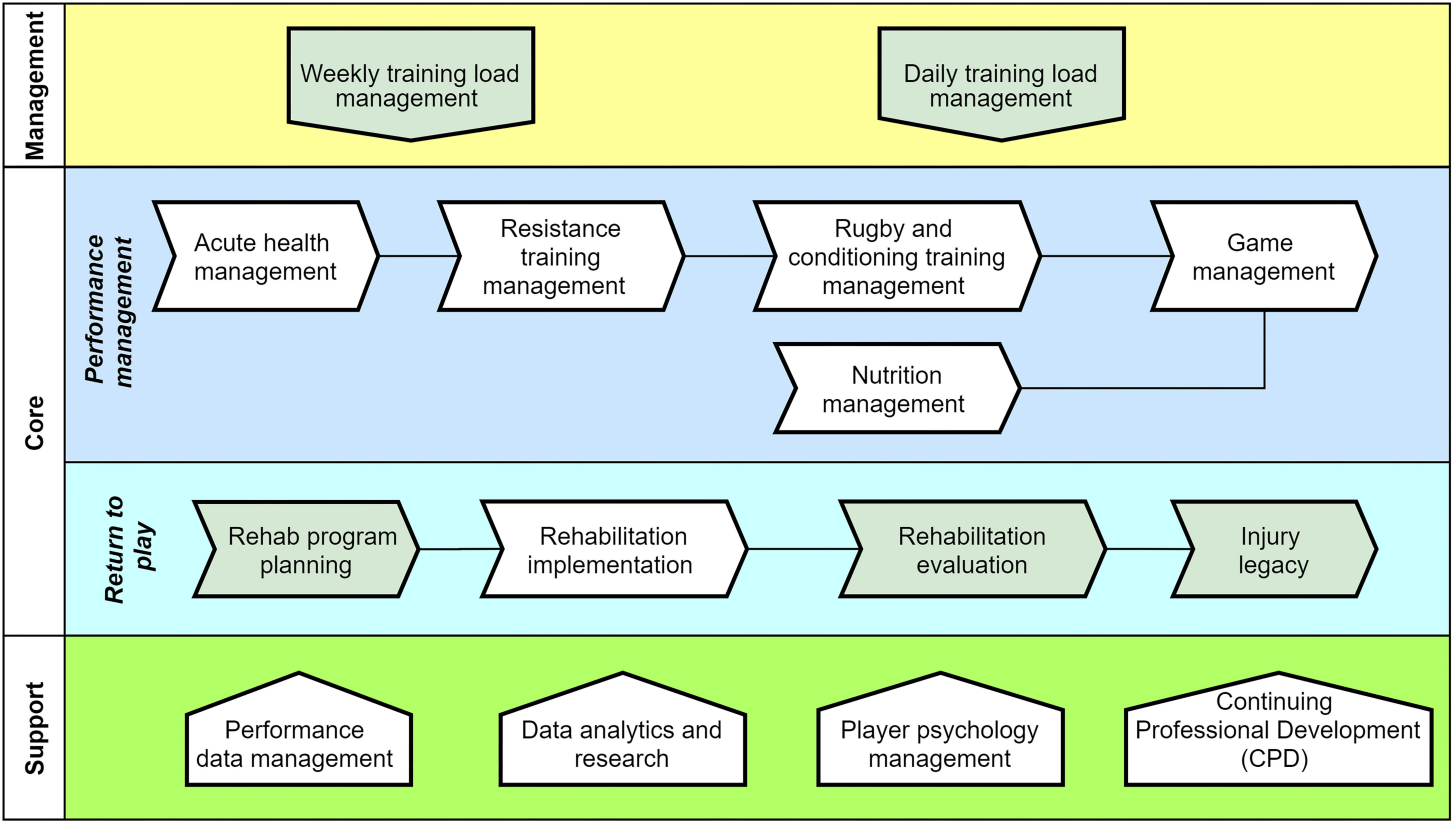
High-Performance Unit process landscape model.

- *High-level processes*: Table 2 illustrates the identified high-level core, management, and support processes executing in the HPU.
- *Value chains*: There were two core value chains identified within the player management processes in the HPU with each illustrating a full distinctive cycle of activities performed by staff to manage the athletes. Those were as follows:
  1. Performance management: As illustrated in Figures 2 and 3, the core in-season value chain that was executed focused on a match scheduled during the weekend (in most cases).
  2. Return to play: The second core value chain (refer to Figure 3) specifically focused on managing injured players and their return to play programs.
- *Process relationships:* The relationships between the core processes in the performance management value chain were defined to identify the lower level sub-process units. For example, as illustrated in Figure 2, the resistance training management process was further decomposed into three lower level sub-processes (resistance training planning, resistance training implementation, and resistance training evaluation) that were executed in sequence. Additionally, the decomposed lower level sub-processes also contained multiple process variants. For instance, the resistance training planning sub-process contained two process specializations (team training planning and individual training planning). As illustrated in Figure 3, the return to play core value chain only contained a sequencing relationship.
- *Process landscape model:* The high-level architecture of the player management processes (including their value chains) identified in the HPU was illustrated using the process landscape model depicted in Figure 3. Among the process units (core and management), the ones generating a decision outcome were selected for further analysis. Those identified processes are listed in Table 3 and are depicted by a “green” color background in Figures 2, 3.

**Table 2:** High-level processes in the HPU (grouped by the three process categories).

**Process category**	**Process name**
Management	Weekly training load management
	Daily training load management
Core	Acute health management
	Resistance training management
	Rugby and conditioning training management
	Nutrition management
	Rehabilitation program planning
	Rehabilitation implementation
	Rehabilitation evaluation
	Injury legacy
	Game management
Support	Performance data management
	Data analytics and research
	Psychology management
	Continuing professional development (CPD)

**Table 3:** Process prioritization ratings.

**ID**	**Process**	**Process category**	**Parameter**						
			**Strategic importance**		**Health**		**Feasibility**		
			**High**	**Low**	**Good**	**Poor**	**Low**	**Medium**	**High**
M1	Weekly training load management	Management							
M2	Daily training load management	Management							
C1	Acute health assessment	Core							
C2	Team resistance training planning	Core							
C3	Individual resistance training planning	Core							
C4	Resistance training evaluation	Core							
C5	Rugby conditioning design	Core							
C6	On-feet conditioning design	Core							
C7	Off-feet conditioning design	Core							
C8	Rugby and conditioning evaluation	Core							
C9	Daily fuelling planning	Core							
C10	Weekly fuelling planning	Core							
C11	Leaner planning	Core							
C12	Gainer planning	Core							
C13	Maintainer planning	Core							
C14	Rehab program planning	Core							
C15	Rehabilitation evaluation	Core							
C16	Injury legacy	Core							

#### Process Selection

From the previous steps, 16 core and two management processes generating a decision outcome were identified for deeper investigation (Table 3). From them, as presented in Table 3, to select the highest priority processes for discovering the information flow on the micro-level, the HPU management team collectively agreed on a rating (subjective) for each process regarding its current strategic importance, health (current information usage), and feasibility for change or optimisation.

**Figure 4:**
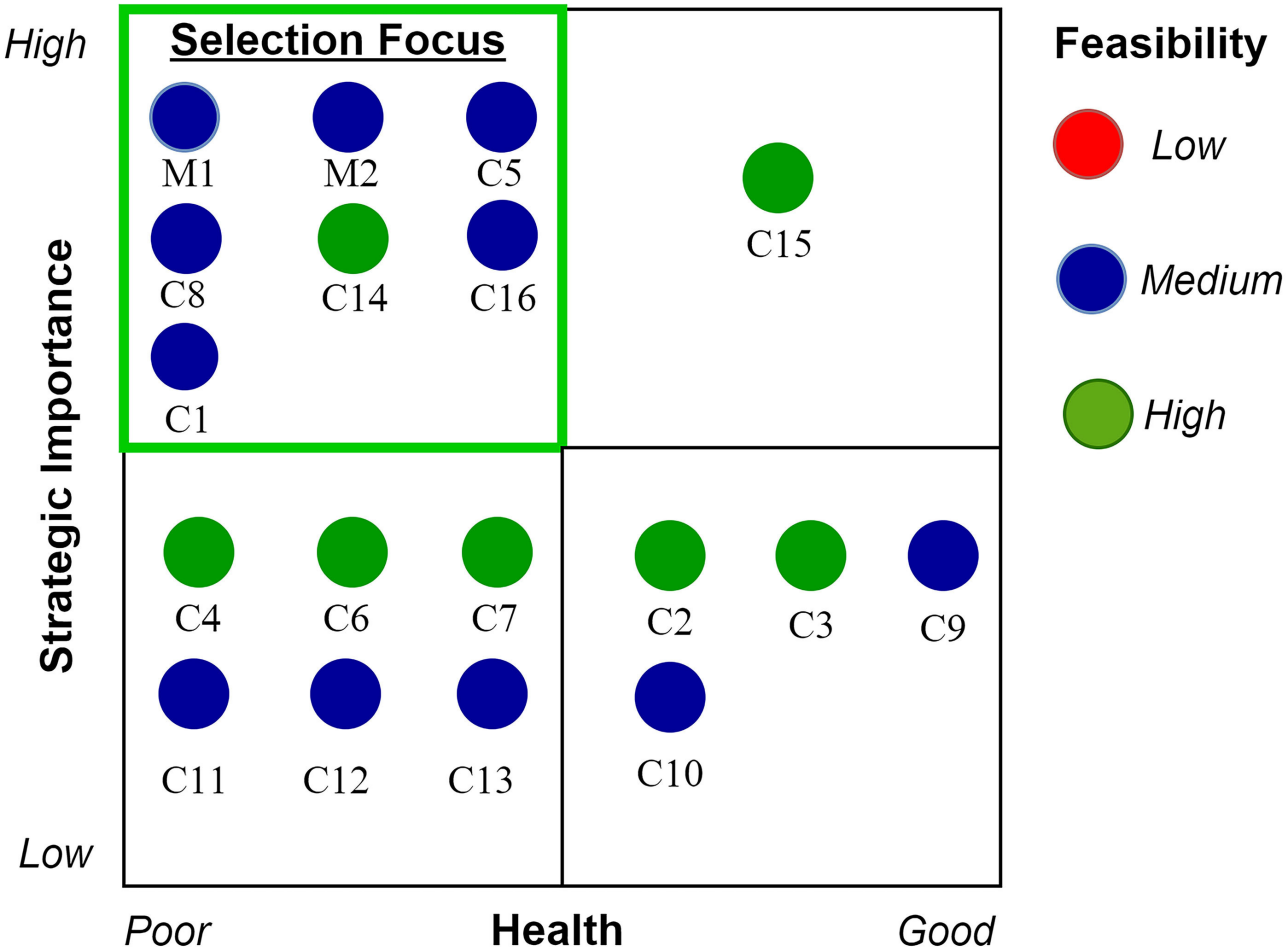
Process portfolio of identified processes for prioritization.

From the management-rated process list, the process portfolio diagram illustrated in Figure 4 was derived, and seven processes (M1, M2, C1, C5, C8, C14, and C16) rated as high current strategic importance, poor information health, and medium or high feasibility were selected for discovering the current issues existing in the information flows associated with them.

### Process Discovery

#### Data Collection

For the selected 7 player management processes, the sponsor (head of applied science and research) defined one other member of the HPU management team as the subject-matter expert (SME) of the weekly training load management (M1), daily training load management (M2), rugby conditioning design (C5), and rugby conditioning evaluation (C8) processes. Additionally, two physiotherapists were defined as the SMEs of acute health assessment (C1), rehab program planning (C14), and injury legacy (C16) processes (refer to Table 3). This resulted in conducting the semi-structured interview sessions (Table 4) to collect the necessary information to model the current state information flow of the selected seven processes (including two interviews with a sports scientist and strength and conditioning coach for collecting additional information). Each process discovery interview lasted <30 min, and Table 4 provides details on the participant characteristics of those interviews.

**Table 4:** Participant characteristics (SME) of the interviews conducted to unravel the current state information flow of the M1, M2, C1, C5, C8, C14, and C16 processes.

**Number of interviews**	**Focused process**	**Subject matter expert (SME)**	**Age**	**Years of experience in professional sport**
2	M1, M2, C5, C8	Member of the HPU management team	39	14
2	C1, C14, C16	Physiotherapist	31	6
1	M1	Sports scientist	27	4
1	M1	Strength and conditioning coach	46	12

**Figure 5:**
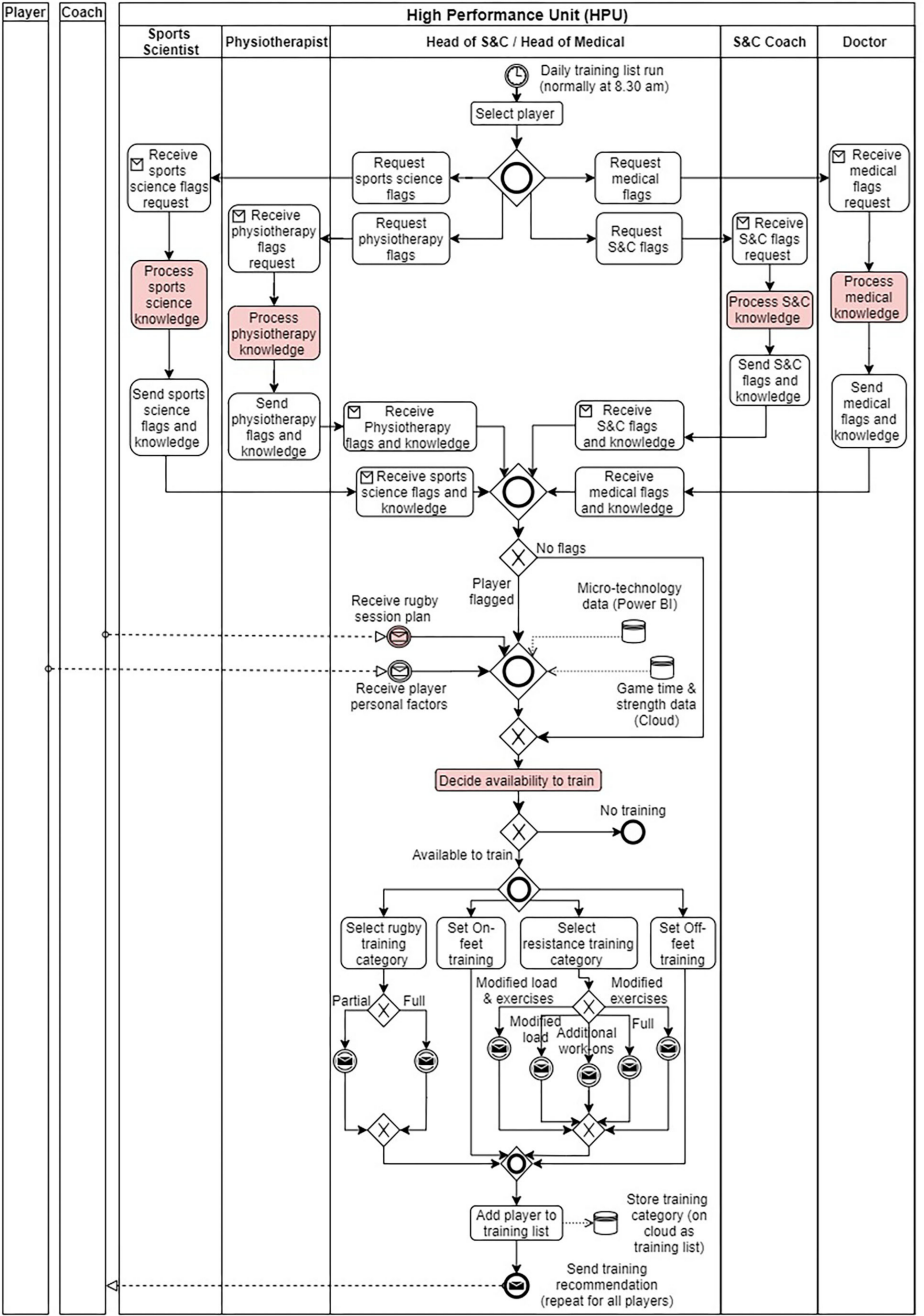
Daily training load management (M2) process As-Is state BPMN model. Tasks and events with potential information issues are in a red color background (discussed under Process Analysis).

For illustration, the article will elaborate on the results obtained from evaluating the information flow of the daily training load management (M2) process. It was the most critical process occurring within the HPU since it generated the daily training recommendation for each player in the squad. Table 5 shows the data collected relating to the M2 process from the interviews with the SME. Next, as illustrated in Figure 5, from the collected information, the current (As-Is) state of the M2 process (including the information flow) was modeled using BPMN.

**Table 5:** Data collected to model As-Is state of daily training load management (M2) process.

**Process name**	**Daily training load management**
Process owner	Head of Medical/Head of Strength and Conditioning
Process objective	To optimize daily physical and rugby stimulus for each player
Trigger events	HPU list run (a daily planned meeting)/coaching white board
Actors	All members of HPU/coaches
Information suppliers	All members of HPU/coaches
Information inputs	Physiotherapy knowledge/strength and conditioning knowledge/sports science knowledge/micro-technology data metrices/rugby training session plan/player factors/game time/strength data
Process steps	Select player
	Request for any flags regarding the player
	If flagged, request information inputs regarding the player
	Decide availability to train
	Select rugby training/resistance training/on-feet/off-feet training categories
	Create training list
	Share training list with staff
Information outputs	Training list (person involved, what he is doing on a day)
Main customer	All members of HPU/coaches
Customer expectation	Understand the training plan of each player for that specific day
Process performance measure	None
Technology	Microsoft Excel, Word, R and Power BI
Data management	SharePoint/Laptop

#### Current State (As-Is) Process Models

**Figure 6:**
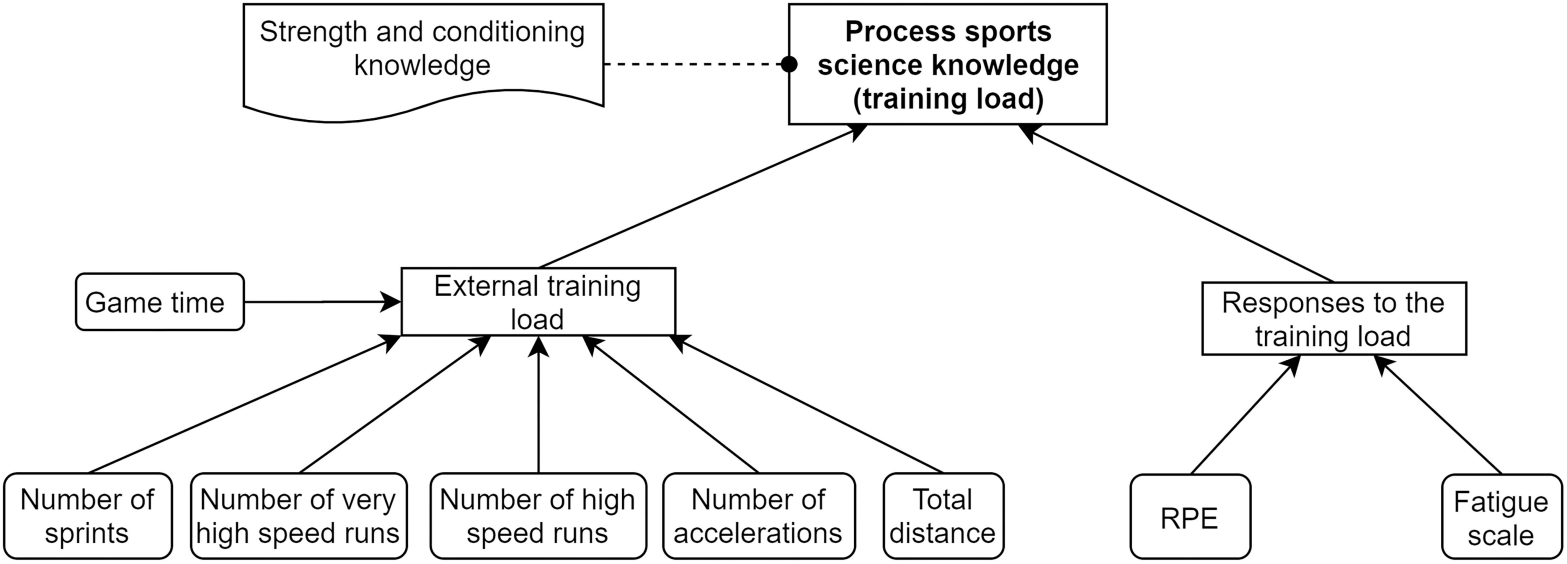
The decision requirement diagram (DMN) of the process sports science knowledge decision point within the As-Is state of daily training load management (M2) process model depicted in Figure 5. Note that the data inputs (except game time) contributing to the external training load decision were generated from micro-technology (GPS).

Figure 5 illustrates the current process execution sequence of the daily training load management (M2) decision process. Specifically, the model illustrates how the daily training recommendation from the HPU was generated for a single player in a normal operating scenario. Initially, after selecting a player, the process owner (head of S&C/head of medical) requested feedback regarding any flags (notifications) from the previous training day for the four key areas of focus in the HPU (sports science, physiotherapy, strength, and conditioning and doctor referral). For example, if the player was flagged for high training load, the sports scientist provided knowledge regarding this flag (derived from currently available micro-technology data sources to the sports scientist, and the specifics of this decision are provided in Figure 6). For flagged players, external information sources such as rugby session plans and player personal factors were also considered when managing the daily training loads. Additionally, player game time and global positioning system (GPS)-based data metrices (e.g., number of high-speed runs) were available as accessible data sources to further investigate the flagged player prior to recommending his daily training category. Based on all the assessed factors, the specific categories for daily training among resistance training, rugby training, on-feet training, and off-feet training were selected for the player. This process was repeated for each player in the squad, and a finalized daily player training recommendation list from the HPU was shared with the coaches.

Additionally, Figure 6 (based on a decision requirement diagram from DMN) illustrates the micro-level interaction between data, information, and knowledge when the sports scientist generated the requested sports science knowledge and flag within the daily training load management process depicted in Figure 5 (refer to the *Process sports science knowledge* activity in a red color background). The core outcome of this decision was to provide knowledge relating to the training loads experienced by the players, which was taken into consideration when generating the final daily training recommendation as an outcome of the M2 process.

### Process Analysis

#### Stakeholder Analysis

To analyse the improvement opportunities in the current state of the daily training load management (M2) process information flow, three focus group discussion sessions were conducted with the main stakeholder groups of the M2 process. Table 6 illustrates the characteristics of the participants in the focus group sessions. An additional unstructured interview with the head of applied science and research was also conducted to elicit issues from the sports science stakeholder perspective.

**Table 6:** Participant characteristics (focus groups) for analyzing M2 decision process.

**Focus group**	**Stakeholders**	**Number of participants**	**Age**	**Years of experience in professional sport**
1	HPU management team	2	35	8
			37	11
2	Strength and conditioning coaches	2	46	12
			27	5
3	Physiotherapists + HPU management team	7	31	6
			31	4
			29	8
			27	2
			29	3
			35	8
			37	11

First, at the onset of each focus group discussion, the relevant stakeholders verified the semantic quality of the As-Is process models. The initial model of the M2 process illustrated in Figure 5 did not contain a separate swim lane for inputs by the doctor (e.g., flagging on concussion review). This lapse in semantic model quality was highlighted during the first focus group session with the HPU management team.

#### Issues in the Current Information Flow

Thematic analysis of the data collected from the focus groups to analyse the As-Is state of the daily training load management (M2) process yielded issues in the current information flow pertaining to two key themes, namely knowledge creation and process flexibility. Each of the main themes contained sub-themes, which highlighted specific issues in the current information flow associated with the M2 process.

- *Knowledge creation:* This theme focused on identifying current issues in the M2 process when generating knowledge from data, information, and evidence. Specifically, it highlighted the current issues associated with the information flow when strength and conditioning coaches, physiotherapists, sports scientists, and doctors accessed data sources and transitioned them to knowledge to generate a decision pertaining to player management (e.g., flagging). As specified in the following text, this theme consisted of five sub-themes that illustrated different current issues in the information flow.
  ○ *Inaccessible data:* Although certain data sources of players such as resistance training and baseline testing data were collected during training, they were not accessible to the practitioners in certain instances. For example, a strength and conditioning coach stated the following regarding resistance training data:

“*If I am sat at home in an evening and you get some information come through and then you want to cross reference it from er from er what they done in a gym perspective all the information (player resistance data) is either on a folder here (training center) or on a whiteboard here (training center)”*

**Figure 7:**
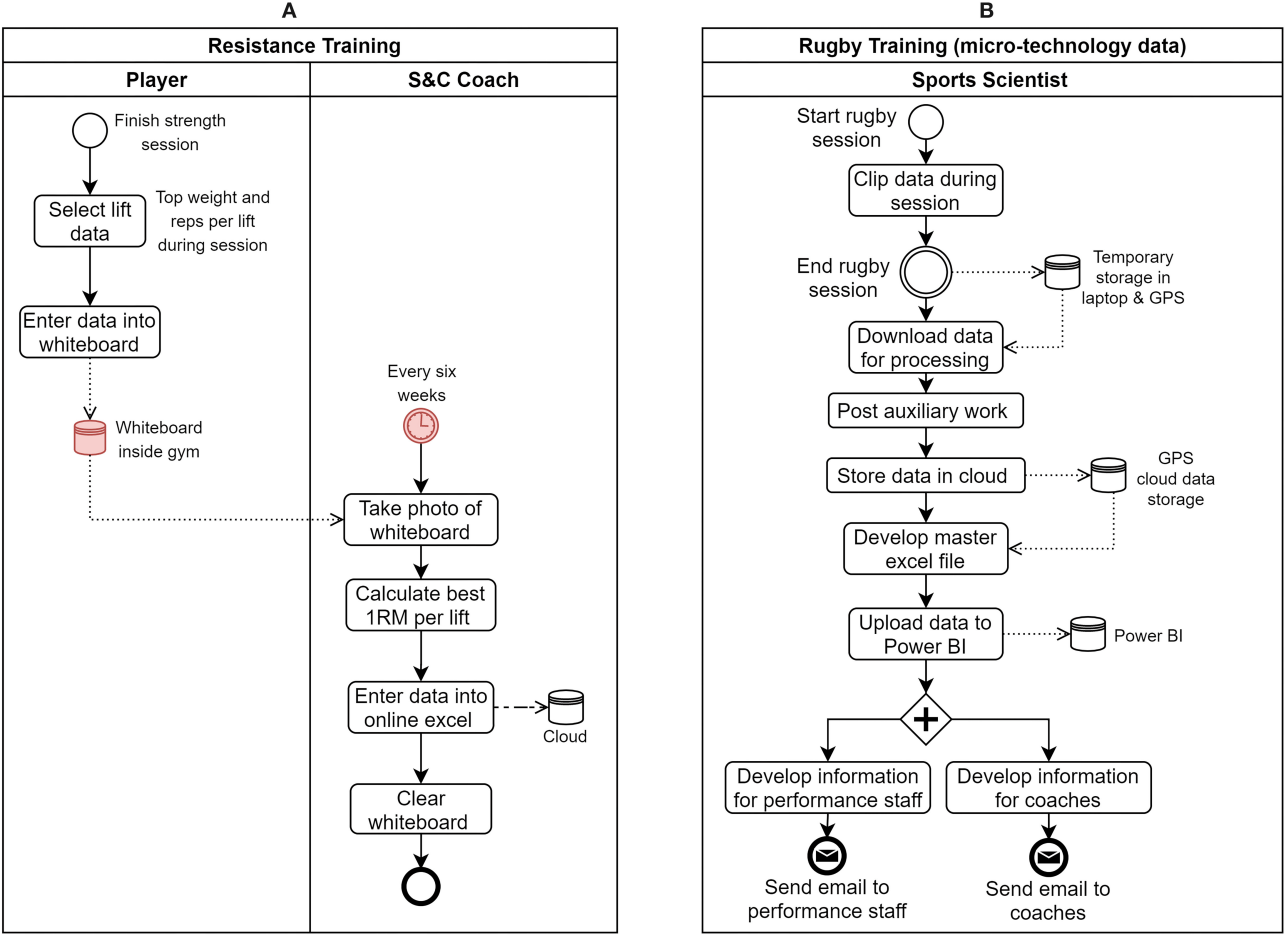
As-Is state process models of **(A)** resistance training (potential issue points contain a red background) and **(B)** rugby training micro-technology data collection processes.

This latter inaccessibility of daily player resistance training data was evident from the current state data collection BPMN process diagram depicted in Figure 7A. In the current state, a player completes a resistance training session and enters his best weights and reps (per lift) into a whiteboard in the gym. Afterward, once every 6 weeks, the S&C coach takes a picture of the whiteboard and calculates the best estimated 1RM (one-repetition maximum) value of each player (per lift) during the 6-week block and enters that data into an online Microsoft Excel sheet. Therefore, the only mode to access daily resistance training data of a player in the current state is by observing the whiteboard in the gym. On the contrary, as illustrated in Figure 7B, daily rugby training GPS-based data metrics of players were accessible as data/information sources on any given instance since the data are processed into a power BI interface to be accessed by the staff.

○ *Unavailable data:* This sub-theme highlighted the unavailability of certain player data parameters for decision-making. However, HPU practitioners were aware that those data sources were more “nice-to-have,” rather than being highly critical for managing players in the current context. As stated by a member of the HPU management team,

“*I've always found S*&*C monitoring fascinating you know and then I think you can get as much from interpreting most of the humans as there is something there you know that's the big outcome fascinated by that you know whether that be through erm objective measurement of er jump height or er erm bar velocity or et cetera et cetera”*

○ *Information noise:* This issue sub-theme mainly focused on HPU stakeholders specifying that the information inputs into the daily training load management (M2) decision process (e.g. rugby session plan) may be incomplete, inconsistent, or late. According to HPU staff,

“*Having the session plan come through from the coaches that's probably one that I think is still not quite getting right consistently in terms of like having the information available it's like concrete in terms of session plan is quite few times where it's all like you know this is we talk through the session in theory what it is and then it's not that”*

- However, HPU practitioners were mindful of the fact that the output from the M2 process (player training list recommendation) may be necessary for coaches to create a finalized session plan. Therefore, the sub-theme further highlighted the issue associated with the improper synchronization of daily training load management (M2) and rugby conditioning design (C5) processes. From the HPU management team's perspective,

“*The session plan that comes through and yet that has that session plan hasn't been practical to adjust to who is available for the session it can have designed the session which is it's not gonna work because the information hasn't been fed up on the availability (player training recommendations generated from M2 process)”*

○ *Unavailable information:* Specifies the unavailability of weekly strategic objectives defined from rugby coaches' perspectives to guide HPU practice and macro-level strategic individual player development goals and targets. For example,

“*I think the retrieval of information from to understand rugby context better whether it's acutely with a session plan or acutely with what bodies you need on deck and what points in the in the rugby training session it needs certain people to longer term vs. er er longer term picture of where the person sits in the depth what's their development plan where where do the coaches see them needing more or less input that's probably information that's hard for us to currently receive”*

○ *HPU flagging consensus:* The management team of the HPU highlighted the importance of having a consensus among the practitioners on the different flags (notifications) generated during player management. Furthermore, the specific data/information input conditions associated with those flags were also not currently available. This illustrated the existence of noise in decision-making relating to the flagging of players in the current context.

“*That we actually need to come to some sort of HPU consensus on this side of stuff (flagging) rather than me just making the decisions the decision will be made potentially by me but it must be made using the consensus of the HPU as to what we believe is right”*

- *Process flexibility:* This major theme specified issues in the current information flow associated with the dynamic and unpredictable nature of player management processes. Specifically, a major sub-theme was the issues in the current information flow associated with incomplete decisions pertaining to player management.
  - ○ *Incomplete decisions:* The current information architecture does not contain a concise information flow to manage scenarios of HPU practitioners being unable to make concrete decisions about the daily training categories of a player within the M2 process. The instances where HPU staff required additional time to observe a player in the morning to recommend his ideal daily training category are not well-supported with an information flow.

“*We (medical team) erm suggest that more time may be needed to see how that clinical entity addresses one way or another and then we apply our knowledge and say well we need more time for that so that it does it it's not until one o'clock we've actually made a sound solid decision on what we're gonna do with that guy”*

“*Given the nature of our game we always have a few guys who are hanging on (incomplete decision from M2 process) and rightly so medical team just gives them more time to see how they try to train”*

## Discussion

The present study aimed to evaluate the current state of the information flow of player management processes occurring within the High-Performance Unit (HPU) at a professional rugby union club in England. The process architecture of the HPU consisted of two core process value chains (performance management and return to play) in normal operating conditions. On the specific level, 18 processes (core and management) that generated a decision outcome were executed to manage the players. From the list of identified processes, the HPU management team prioritized 7 processes for analyzing the current state of their information flows. Semi-structured interviews with the subject-matter experts of those processes yielded the As-Is state BPMN process models (Figures 5–7) that highlighted the current information interaction during process execution. The results pertaining to the information flow issues existing in the daily training load management (M2) process were presented in the article. Specifically, two main themes (knowledge creation and process flexibility), decomposing to six sub-themes of current issues existing in the information flow of the M2 process were derived from the thematic analysis conducted on the data obtained from three focus group discussions with the main stakeholder groups of the M2 process.

### Process Architecture

The derived value-chain of the player management process architecture in the HPU was based on the normal operating conditions during the in-season of the Gallagher Premiership. Hence, the identified process value chain during the week was shaped toward a game scheduled during the weekend and was linked based on a daily operational sequence (e.g., acute health management occurred prior to the resistance training management process). Although the pre-season value chains within a week would still resemble the process landscape model depicted in Figure 3, the focus of the pathway would incline more toward the starting game of the new season. The specifically formulated process architecture in the current study is unique to this organization. However, practitioners could use the methods used in the article to formulate the process architecture in their environments to assist in identifying the current issues in the information flows associated with the player management processes.

In the current case study, processes for discovering the micro-level information flow were selected subjectively based on the management perspective. However, if KPIs of processes that relate to an information flow (e.g., time to notify a soft tissue injury) and specific thresholds of poor performance of those KPIs were available in a considered environment, practitioners could use such measures to objectively select processes for exploring current issues in their information flows based the KPI values.

### Current State (As-Is) Process

Interview-based process discovery was well-suited to unravel in-depth details on the current state of the identified player management processes due to two main reasons: (1) The nominated SMEs had vast knowledge of the current state of the selected processes since they were the main individuals responsible for executing them. (2) Other methods like automatic process discovery (e.g., based on event logs) were not suitable in the current rugby union environments since player management processes were executed based on human intuition, rather than system automation. However, in most situations, the SMEs were inclined to provide knowledge of the normal operating situation of a process. Therefore, as the analyst, author JR had to specifically discuss abnormal process scenarios to gather details on how the SMEs executed the processes during unconventional situations.

The As-Is state process models developed from BPMN and decision models from DMN standards clearly illustrated how data and information flowed through the considered decision processes. Specifically, process models with swim lanes (i.e., delineating who does what in a process) corresponding to each stakeholder of the process demonstrated how different individuals interacted with the information and highlighted the specific points at which knowledge was generated during process execution. Additionally, having the As-Is state of the process as a model greatly supported the process analysis focus group discussions since the stakeholders were able to refer to it when elaborating on current state issues, rather than building verbalized visuals for the participants. This latter aspect was achievable since the syntactic representations of BPMN models were easily understood by the participants.

However, it must be stated that process models like those derived from standards like BPMN and DMN are typically used to optimize automated processes. This is because normally, such workflow models are directly transformed to executable form and implemented as automation using information technology systems like Business Process Management Systems (BPMS). Whilst that is the common case, it does not limit process modeling techniques or BPM as an overall framework to be used to model or optimize just automated processes. Instead, as supported by the definition of BPM by the Association of Business Process Management Professionals (ABPMP), BPM as a change management tool and its inherited modeling techniques can be equally used to optimize non-automated processes as well (ABPMP, 2019). The results from the current study further support the latter statement and the resultant use of standards like BPMN and DMN as valuable approaches to model non-automated processes like those found in professional sporting environments. Therefore, in the current study, a syntactic quality assessment of the workflow models was not conducted since the goal of process modeling was to provide a reference to understand current execution steps and existing issues, rather than transforming the models into a system engine like a BPMS.

### Current Issues in the Information Flow

In reference to the daily training load management (M2) process, daily resistance training and baseline testing data were the major inaccessible data sources for decision-making. Apart from the latter two data sources, the thematic analysis further illustrated that HPU practitioners were generally content with the currently available raw data sources. The practitioners had a positive interaction with GPS data and were readily accessible to HPU staff during decision-making. Whilst GPS data were optimized in terms of availability, there is still space to explore the state of other information quality dimensions (e.g., timeliness, relevancy, free of error, and completeness) pertaining to GPS-based measurements. However, the analysis of processes with more coaching interaction like rugby conditioning design (C5) highlighted three data metrics that strongly aligned with the coaching/game strategy of the club and were currently unavailable. Those latter data parameters were not discussed in the article due to intellectual property restrictions.

Moreover, the identified issues in the current state of information flow illustrated that the fundamental needs for optimisation within the considered case study environment existed in the transitioning from data to information (e.g., inaccessible data and information noise) and information to evidence (e.g., flagging consensus). Resultantly, from an optimisation perspective, typical digitization endeavors like data architecture development (e.g., ETL/ELT pipelines) (Blobel and Lames, 2020) and data visualizations (Perin et al., 2018) that are increasingly being utilized in sporting environments appear to be applicable techniques to improve the data to information transition in the considered environment. However, reverting back to Dammann's (2018) framework signifies that improving the information to evidence transformation would require the establishment of necessary standards that could be used to generate evidence from the information. We feel that the development of such standards requires deeper scientific exploration as they may be generated as an amalgamation of subjective (e.g., practitioner intuition) and objective (e.g., data analytics models) perspectives. Finally, information flow issues like late rugby session plans highlighted the dynamic and non-rigid nature of decision-making within professional rugby union environments. Such features were the main reason for the synchronization issues which existed in the information flow.

## Conclusion

Practical case studies exploring the optimisation of information flows required for rugby union player management is lacking in the current sports literature. This article, based on a case study from a professional rugby union environment, presented the results from the first part of an attempt to fill this gap in the sports literature by demonstrating how practitioners could evaluate the current information flow and identify specific issues or gaps associated with player management processes. Overall, the BPM lifecycle used in the study was successful in identifying issues in the information flow of the considered sporting environment. Although the outcomes from stakeholder analysis highlighted that the major issues in the information flow associated with rugby union player management may exist within the process of transforming data to knowledge, we do not wish to generalize this statement since the operating dynamics of each rugby union club may be unique. However, the stepwise methods described in the article to unravel existing issues in the information flow can be used in any team sports environment.

In conclusion, the information flow relating to professional rugby union player management processes may contain misalignments to organizational objectives and specific issues or gaps that require attention. Whilst the rapidly growing trend of developing analytical models for analyzing player performance from available data is appealing and beneficial, there may be gaps in the current information flow which feed into such analytical models or wider decision processes in sporting environments. Therefore, sporting organizations could reap great benefits by initially identifying such issues in the operational information flow and optimizing them prior to concentrating heavily on analytics. A failure to bridge such gaps in the information flow and align them to the organizational goals will generate the risk of developing models on sub-optimal information flows, which may not support the governing strategies of an organization. Finally, through a separate article (Ranaweera et al., 2022), we have presented how the data to information transitioning of the HPU information flow was optimized by using the process redesign, implementation, and monitoring phases of the BPM lifecycle.

## Data Availability Statement

The original contributions presented in the study are included in the article/supplementary material, further inquiries can be directed to the corresponding author/s.

## Ethics Statement

The studies involving human participants were reviewed and approved by Local Research Ethics Co-ordinator (LREC), Carnegie School of Sport, Leeds Beckett University. The patients/participants provided their written informed consent to participate in this study.

## Author Contributions

JR, DW, and GR conceptualized the study design and contributed to writing and editing the manuscript. JR, MZ, and GR participated in data collection. JR performed the initial data analysis. DW, MZ, and GR validated the resulting outcomes. All authors have read and approved the final version of the manuscript.

## Conflict of Interest

The authors declare that the research was conducted in the absence of any commercial or financial relationships that could be construed as a potential conflict of interest.

## Publisher's Note

All claims expressed in this article are solely those of the authors and do not necessarily represent those of their affiliated organizations, or those of the publisher, the editors and the reviewers. Any product that may be evaluated in this article, or claim that may be made by its manufacturer, is not guaranteed or endorsed by the publisher.
